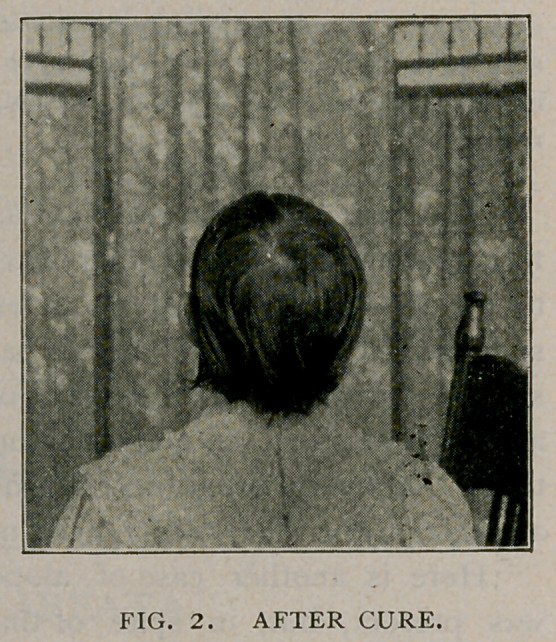# Alopecia Areata

**Published:** 1898-11

**Authors:** Joseph Spangenthal

**Affiliations:** Buffalo, N. Y., Physician for diseases of the skin at the German Hospital Dispensary; visiting physician to City Hospital for Women


### Clinical Reports.

### ALOPECIA AREATA.

By JOSEPH SPANGENTHAL, M. D., Buffalo, N. Y.,
Physician for diseases of the skin at the German Hospital Dispensary; visiting physician to City Hospital for Women.

THE etiology of alopecia areata is so imperfect that the disease must necessarily be placed among those whose etiology is classified as obscure. By a conservative dermatologist, it is considered a noncontagious, trophoneurotic condition, accompanied by an atrophy of the hair follicles and even of the hair itself. It has its origin in a nervous influence, produced by the direct action of a functional nerve disturbance of the hair follicle. Some believe that the affection should be placed among the parasitical, and that it is caused by a fungus, like that of the tinea tonsurans; while others suggest that there is a possible connection between the two diseases. The parasitic theory finds support in the occurrence of epidemics, which necessarily imply a contagious element. In this country one epidemic of apparently alopecia areata has been reported by Putnam in a paper published in the *Archives Pediatrics*, 1892, IX., p. 595. This theory is further supported by the discovery of a micro-bacillus by Sabourad.

The case here reported was especially due to an injury received by the patient, as stated below:

Miss H., aged 22; seamstress; presented herself for treatment at the skin clinic, University of Buffalo, service of Dr. Grover Wende, last year, with the following history: While sewing before a window she was struck by a ball, which some one had carelessly tossed through it, causing considerable inconvenience for two or three days. Nine days following she noticed a thinning of her hair, finally resulting in a bald spot over the left parietal bone. Six weeks later she developed a complete alopecia of the scalp. All this occurred without pain or subjective disturbance of any kind. The patient was given the usual remedies in the form of local applications and within six months her hair was fully restored.

This case must be regarded as a typical one, being clearly due to nervous shock and distinguished from cases which can be traced to a microörganism. Many cases have been reported which were supposed to be due to various causes, such as grief, fright, mental shock, operations on the neck, or to influences much less rational as, too little sleep, an unsatisfactory hygienic condition or malnutrition. Recovery often occurs in these cases without the usual application for the destruction of microörganisms.

Here is another case of alopecia areata where trophoneurosis was present, but, in spite of this fact, the affection manifested itself in the form of round patches which increased in size by peripheral extension, strongly suggesting the cause of this particular condition. It would, of course, be hard to determine the exact cause, since, according to the parasitic theory, the nervous condition might be only a predisposing factor in the case:

Figure 1 represents a case of alopecia areata, as follows:

Josephine R., aged 14; had six brothers and sisters, two of whom died from convulsions; had no sickness during her childhood; both parents affected by rheumatism and heart disease. At the age of 10 she began to have choreic movements, which constantly grew worse until she developed a marked case of the disease, the acute symptoms lasting for six months, her condition being at one time so severe as to affect her speech. At the expiration of that time her condition improved and her chorea was decidedly better; however, her muscular movements have never become natural. She is still very nervous at times. About one year ago, some two and a half years following the beginning of the chorea, she would occasionally, during the day, become so agitated that her mother feared that she was about to fall a victim to her old trouble. At this time she made her first visit to the University dispensary, exhibiting a bald spot, which had begun to develop a few weeks before on the occipital region. This was soon followed by others, which coalesced, causing a large patch. This continued to spread until the top of the scalp was entirely denuded. About this time another bald patch appeared upon the right side, at the junction of the non-hairy parts, and after a time the disease attacked the left side, gradually extending around the head, until almost three-fourths of the scalp was denuded, the result showing the form of a band. Heart and lungs normal, urine revealing neither albumin nor sugar, but an abundance of oxalate of calcium crystals. At the time the patient was seen there was prescribed an ointment of chrysarobin a half dram, adeps one ounce. This created considerable irritation of the skin without producing any beneficial effects. Numerous other lotions were prescribed, but, as a rule, failed in their

FIG. I. BEFORE TREATMENT.

FIG. 2. AFTER CURE.

purpose. Bichloride of mercury was then tried, one grain to an ounce. This seemed to stop the hair from falling, whether through its irritating or its anti-parasitic virtue remains to be known. The amount of bichloride was gradually increased for a time when the following prescription was made and is still in use:

Hydrarg. bichlor.................................gr. xx.

Glycerine ....................................... ʒ iv.

Aqua cologniensis............................... ℥ xviii.

Under this treatment, not only did the baldness cease to spread, but fine downy hair began to spring up all over the bald patches. This at first was white, but finally the growth became more vigorous, the pigment returned and in about twelve months after the commencement of the disease the patient had fully recovered, presenting the appearance shown in Figure No. 2.

629 MAIN STREET.

## Figures and Tables

**FIG. I. f1:**
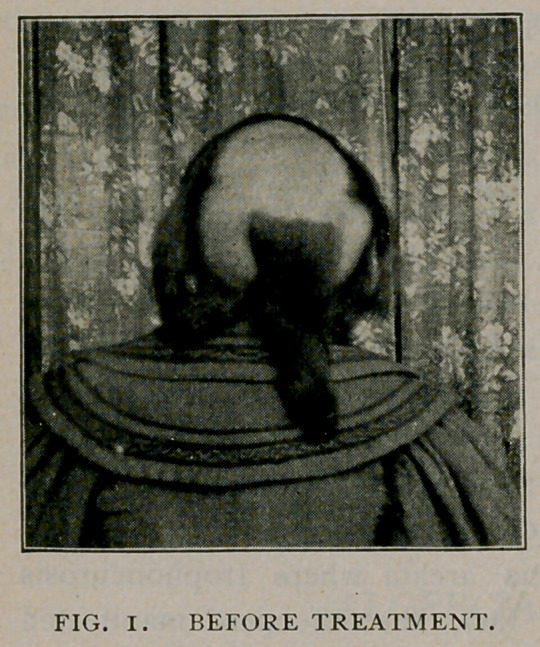


**FIG. 2. f2:**